# Unraveling Immune Checkpoint Inhibitor-Associated Cardiovascular Toxicities: Pathway Insights, Mechanisms, and Emerging Therapeutic Targets

**DOI:** 10.31083/RCM43846

**Published:** 2026-02-10

**Authors:** Biqi Zhang, Mairedan Muhetarijiang, Xiangjie Sun, Yue Wu, Ryan Justin, Zhoubin Li, Ting Chen, Dongchen Zhou, Xiaosheng Hu

**Affiliations:** ^1^Department of Cardiology, The First Affiliated Hospital, College of Medicine, Zhejiang University, 310003 Hangzhou, Zhejiang, China; ^2^School of Medicine, Zhejiang University, 310058 Hangzhou, Zhejiang, China; ^3^Department of Lung Transplantation and General Thoracic Surgery, The First Affiliated Hospital, College of Medicine, Zhejiang University, 310003 Hangzhou, Zhejiang, China; ^4^Key Laboratory of Precision Medicine for Atherosclerotic Diseases of Zhejiang Province, Affiliated First Hospital of Ningbo University, 315010 Ningbo, Zhejiang, China

**Keywords:** immune checkpoint inhibitor, cardiovascular toxicity, molecular mechanism, signaling pathway

## Abstract

Immune checkpoints are critical regulatory molecules in the immune system that maintain self-tolerance by preventing excessive immune activation against healthy tissues while being exploited by malignant cells to promote tumorigenesis and metastasis through immune evasion mechanisms. Immune checkpoint inhibitors (ICIs), represented by programmed cell death protein-1 (PD-1) inhibitors, are a revolutionary class of antitumor therapeutics that have achieved remarkable clinical success over the last decade, with the application of ICIs expanding to a broader spectrum of malignancies. Nonetheless, the administration of ICIs may induce immune dysregulation, potentially leading to the development of multiple immune-related adverse events (irAEs) across various organ systems. Cardiovascular toxicities are a series of relatively rare but severe irAEs that are drawing increasing attention. This review summarizes the latest findings in immune checkpoint signaling pathways and the potential mechanisms underlying the development of various cardiovascular toxicities associated with immunotherapies. Additionally, we also evaluate advances and novel therapeutic targets in the treatment of cardiovascular toxicities.

## 1. Introduction

Immunotherapy refers to a revolutionary anti-cancer strategy that aims to enhance and utilize the immune response to treat cancer, which has developed rapidly in the last few decades [1, 2]. Immunotherapy includes oncolytic virus infection, anti-cancer vaccination, cytokine treatment, adoptive cell therapies, and immune checkpoint inhibitors (ICIs) [3]. Immune checkpoints stand for molecules acting as brakes in the immune system that can preserve immune tolerance in organisms, which primarily include cytotoxic T lymphocyte-associated molecule-4 (CTLA-4), programmed cell death receptor-1 (PD-1), programmed cell death ligand-1 (PD-L1), Lymphocyte activation gene-3 protein(LAG-3), T-cell immunoglobulin- and mucin-domain-containing molecule (TIM-3), and T cell immunoglobulin and ITIM domain (TIGIT).

Since the cDNA of PD-1 and its ligands, PD-L1 and PD-L2, were isolated at the end of the last century, numerous studies have been conducted to explore the pathophysiological functions of immune checkpoints and their pathways. When PD-1/PD-L1 binding was found to inhibit T-cell activity and enable tumor immune escape [4, 5, 6, 7], scientists began developing immune checkpoint-targeting therapies, bringing new possibilities for cancer treatment. Since the first two PD-1 mAbs, Pembrolizumab and Nivolumab, received approval from the Food and Drug Administration (FDA), ICIs have been applied for non-small-cell lung cancer, Hodgkin lymphoma, urothelial carcinoma, renal cell carcinoma, hepatocellular carcinoma, gastric cancer, etc. [8, 9, 10, 11]. However, despite the broad use of ICIs, the clinical outcome remains unsatisfactory due to the primary or secondary resistance, tumor recurrence, and immune-related adverse events (irAEs) [12]. IrAEs consist of a wide spectrum of complications involving multiple organs, including pneumonia, diarrhea, hepatitis, dermatological and endocrine toxicities, as well as rare adverse events like neurological, ocular, and cardiac toxicities [13, 14, 15]. Though cardiovascular toxicities occupy only a small proportion of the incidence of irAEs, the high lethality and rapid progression have made it a serious concern in immunotherapy [16]. In the following sections, we will thoroughly examine the molecular basis and signaling pathways of various immune checkpoints, explore the mechanisms underlying different immune-related cardiovascular toxicities, and summarize the latest advances in treating these events.

## 2. Pathways and Mechanisms of Immune Checkpoints

### 2.1 PD-1/PD-L1 

PD-1 is a transmembrane glycoprotein comprising an extracellular region, a transmembrane region, and a cytoplasmic tail [1, 17], which is expressed on various kinds of immune cells, especially on T cells [13, 18, 19]. PD-L1, also known as CD274 and B7-H1, contains immunoglobulin variable region (IgV) and immunoglobulin constant region (IgC)-like extracellular domains, a transmembrane region, and a cytoplasmic tail without typical motifs [20], and is widely expressed on cancer cells, epithelial cells, dendritic cells (DCs), etc. In the presence of constant antigenic stimulation, such as autoantigen exposure, chronic virus infection, and tumor infiltration, PD-L1 exhibits a significant inhibitory effect on immune reaction. PD-L1 expression can be induced by exogenous or endogenous factors. Exogenous factors include inflammatory cytokines such as interferon (IFN)-γ, tumor necrosis factor (TNF)-α, interleukin (IL)-2, IL-4, IL-10, IL-17, and IL-27, while endogenous factors involve *Pten* gene loss, *Ras* mutation, *Egfr* mutation, *Eml4-Alk* translocation, and *Myc* gene activation [20, 21].

**Fig. 1.**
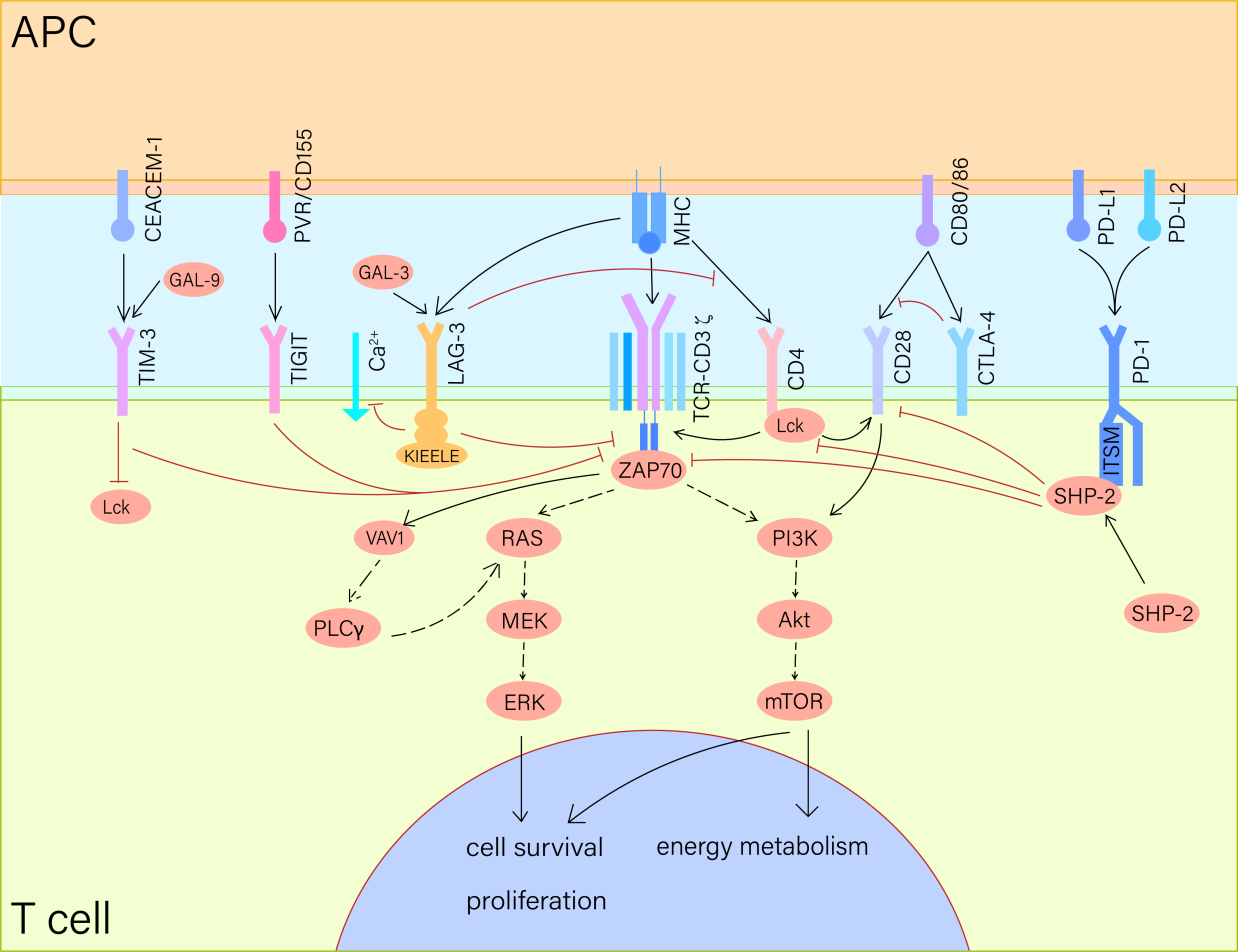
**Immune checkpoint signaling pathways**. When the TCR-CD3 complex recognizes MHC on APC, PD-1 binds to PD-L1 to aggregate SHP-2, which phosphorylates the ITSM on PD-1 while dephosphorylating CD28 and downstream molecules like PLCγ and PI3K. SHP-2 can also inhibit the activity of Lck. CTLA-4 interacts with SHP-2 and competes with CD28 to bind CD80 and CD86. LAG-3 binds to the ligand MHC II and competes with CD4, and the KIEELE motifs can directly inhibit TCR signaling and reduce the inflow of calcium ions. TIM-3 binds to CEACAM-1 or GAL-9 and regulates the activity of Lck. The attachment of TIGIT to PVR on APC initiates the phosphorylation of PVR and ERK in DCs. Under the influence of multiple immune checkpoints, the PI3K-Akt-mTOR and the RAS-MEK-ERK pathway are inhibited, leading to enhanced apoptosis, suppressed cell proliferation, and energy metabolism. PVR, poliovirus receptor; CEACAM-1, carcinoembryonic antigen-related cell adhesion molecule 1; GAL-3, galectin 3; GAL-9, galectin 9; Lck, lymphocyte-specific protein tyrosine kinase; ZAP70, Zeta Chain Associated Protein Kinase 70kDa; SHP-2, src homology region 2 domain-containing phosphatase-2; solid arrow, direct interaction; dash arrow, indirect interaction (intermediate molecules omitted); Vav1, Vav Guanine Nucleotide Exchange Factor 1; PLCγ, phospholipase Cγ; RAS, ras protein; MEK, MAPK/ERK kinase; ERK, extracellular regulated protein kinases; PI3K, phosphatidylinositol-3-Kinase; Akt, protein kinase B; mTOR, mammalian target of rapamycin; MHC, major histocompatibility complex; MAPK/ERK, mitogen-activated protein kinase/extracellular signal-regulated kinase; TCR, T cell receptor; APC, antigen-presenting cell; PD-1, programmed cell death receptor-1; PD-L1, programmed cell death ligand-1; ITSM, immunoreceptor tyrosinebased switch motif; DCs, dendritic cells; CTLA-4, cytotoxic T lymphocyte-associated molecule-4; LAG-3,Lymphocyte activation gene-3 protein; TIM-3, T-cell immunoglobulin- and mucin-domaincontaining molecule; TIGIT, T cell immunoglobulin and ITIM domain.

We have summarized the general mechanisms of the PD-1/PD-L1 pathway and its downstream signaling (see Fig. 1). When a T cell recognizes the major histocompatibility complexes (MHCs) on an antigen-presenting cell (APC) and forms a conjunction, PD-1 binds to PD-L1 and translocates to the T cell receptor (TCR) microcluster, which includes TCR and a series of signaling molecules [22]. The binding of PD-1 and its ligand results in the gathering of Src homology region 2 domain-containing phosphatase-2 (SHP-2), a key regulator in the PD-1 pathway. This process is mediated by the immunoreceptor tyrosine-based switch motif (ITSM), one of the tyrosine-based structural motifs on the cytoplasmic tail of PD-1 [23, 24]. The phosphorylation on ITSMs is enhanced by SHP-2 after the ligation of PD-1 and PD-L1, and the colocalization of PD-1 and TCR microcluster can suppress the TCR signaling by dephosphorylating TCR-associated CD-3ζ and ZAP70, as well as the downstream molecules such as Erk, Vav1, and phospholipase Cγ (PLCγ), for instance [1, 22]. The phosphorylated SHP-2 mainly downregulates PI3K-Akt-mTOR and RAS-MEK-ERK pathways, inhibiting the proliferation and activation of T cells. Besides TCR/CD3^+^ signaling, SHP-2 can also dephosphorylate CD28 to attenuate T-cell activation [19, 25]. This mechanism leads to the exhaustion of particularly CD8^+^ T cells, and consequently, the immunosuppression in the tumor microenvironment [1, 25]. Moreover, the PD-1/PD-L1 signaling has also been reported to suppress the secretion of immune factors such as IL-2 and IFN-γ, and alter the energy metabolism in T cells by affecting glycolysis and lipolysis [17]. The PD-1 pathway also affects B cells. Okazaki *et al*. [26] confirmed that the recruitment and phosphorylation of SHP-2 induced by PD-1 signaling can inhibit BCR signaling by dephosphorylating the crucial downstream molecules, including Igβ, Syk, PLCγ2, and PI3K.

As cancer cells present high expression levels of PD-L1, the exhaustion and apoptosis of T cells induced by PD-1 signaling are increased in the tumor microenvironment (TME), leading to impaired immune surveillance. Thus, blocking PD-1/PD-L1 ligation can potentiate T-cell immunity and play an anti-tumor role in cancer therapy [27]. The monoclonal antibodies that target PD-1 can either block PD-1 in TME or the tumor-draining lymph nodes (TDLNs), enhancing the TCR/CD3 and activating T-cell reaction. On one hand, the exhausted CD8^+^ T cells can be directly reinvigorated by PD-1 inhibitors *in situ*, but in a less efficient way. Since the TME is generally considered hypoxic, mitochondrial dysfunction and reactive oxygen species (ROS) overload caused by hypoxia can facilitate the exhaustion of T cells, making them more resistant to PD-1 inhibitors [28]. On the other hand, the PD-1 inhibitors generate stem-like precursor exhausted CD8^+^ T cells in lymph nodes, which can be transported to the tumor site via the CXCR3-CXCL9 axis and converted to effector-like CD8^+^ T cells to fight tumor cells [10, 29]. Ma *et al*. [30] compared the single-cell transcriptome of cardiac tissues from *Ctla4^+⁣/+^Pdcd^-⁣/-^* and *Ctla4^+⁣/-^Pdcd^-⁣/-^* mice, and found that CXCL9^+^/CXCL10^+^ macrophages are significantly upregulated in the latter model, induced by the secretion of IFN-γ from CD8^+^ T cells. This conclusion indicates a feedback loop that CD8^+^ T cells promote the proliferation of CXCL9^+^/CXCL10^+^ macrophages, which then recruit additional CD8^+^ T cells from TDLNs. Additionally, it’s been reported by Chamoto *et al*. [10] that the PD-1 inhibitor-induced CD8^+^ T cells in the TDLNs present elevated mitochondrial activity, featuring higher levels of ROS and increased mitochondrial mass. The PD-1 inhibitors have also been reported to decrease the myeloid-derived suppressor cells (MDSCs) at tumor sites [31]. However, some studies have proposed a different perspective, suggesting that PD-1 inhibitors exert distinct effects on different T cell populations. Kumagai *et al*. [32], by analyzing the exome of cancer cohorts treated with PD-1 inhibitors, proposed that while blocking PD-1 increases the proliferation and pro-inflammatory cytokine production by CD8^+^ T cells, it works oppositely on regulatory T cells (T_reg_ cells) as PI3K-Akt signaling leads to reduction instead of expansion of T_reg_ cells [24]. This study suggests that a higher proportion of CD8^+^ T cells to effector T_reg_ cells in the TME is related to a better outcome in anti-PD-1 therapy.

In conclusion, PD-1 inhibitors improve anti-tumor immunity in the following ways: (a) PD-1 inhibitors can reinvigorate the exhausted CD8^+^ T cells *in situ*. (b) PD-1 inhibitors can function *ex-situ* to promote the recruitment of CD8^+^ T cells to the tumor site from TDLNs. (c) PD-1 inhibitors can drive the production of pro-inflammatory cytokines such as IFN-γ from CD8^+^ T cells. (d) PD-1 inhibitors can indirectly activate CD8^+^ T cells by altering the energy metabolism. Nevertheless, PD-1 inhibitors induce diverse reactions in different immune cells, which implies their unpredictable effect on the organism.

### 2.2 CTLA-4 

CTLA-4, or CD152, is a surface protein found on activated CD4^+^ and CD8^+^ T cells [33]. The activation of naive T cells requires two critical signals: the TCR provides an antigen-specific signal, while a second set of signals from T-cell costimulatory pathways amplifies the response [34, 35, 36]. CTLA-4 and the co-stimulatory receptor CD28 both target B7-1 and B7-2 (CD80, CD86) (see Fig. 1), a pair of co-stimulatory molecules on APCs [37]. CTLA-4 is located in the cytosol in resting T cells, and is translocated to the membrane following TCR engagement and CD28 co-stimulation. Upon reaching the surface, CTLA-4 vies with CD28 for binding to B7-1 and B7-2, which subsequently inhibits CD28-mediated co-stimulatory signals and suppresses T cell proliferation and activation [38, 39]. CTLA-4-deficient mice usually die early due to a severe lymphoproliferative disorder, featuring unchecked polyclonal proliferation of T cells. In the study by Waterhouse *et al*. [40], *Ctla4*^+⁣/-^ mice born normal but *Ctla4*^-⁣/-^ mice exhibited significantly enlarged spleens and lymph nodes, with large amount of activated CD4^+^ and CD8^+^ T cells. Large spleens and lymph nodes were also observed in the study of Tivol *et al*. [41], and meanwhile they also reported abundant CD4^+^ and CD8^+^ T-cell infiltration in the myocardium. These genetic models highlight the importance of CTLA-4 in restraining T cell immunity for the growth and development of the organism.

The mechanistic basis of CTLA-4-mediated inhibitory signaling remains complex due to conflicting evidence and context-dependent signaling outcomes [42]. Several proposed mechanisms include: (a) the extracellular domain of CTLA-4 competes with CD28 for binding to CD80 and CD86, thereby disrupting co-stimulatory signals [43]. (b) CTLA-4 alters the localization of CD28 in the immune synapse [44]. (c) CTLA-4 regulates TCR signaling via SHP-2 and the serine-threonine phosphatase PP2A [45], while also influencing the assembly or integrity of lipid rafts on the T cell surface [36, 46, 47, 48, 49]. These mechanisms collectively contribute to the inhibitory signaling mediated by CTLA-4, reducing T-cell proliferation and activation.

### 2.3 LAG-3

LAG-3, also known as CD223, was first identified in 1990 by Triebel *et al*. [50] as a membrane protein highly related to CD4, which was detected in activated T and natural killer (NK) cells [51]. As relevant studies are carried out, LAG-3 was also reported to be expressed on B cells and plasmacytoid DCs. LAG-3 is composed of an extracellular region comprising four Ig superfamily domains, a transmembrane region, and a cytoplasmic region that consists of three parts: (1) a serine phosphorylation site, (2) a ‘KIEELE’ motif, and (3) a glutamate-proline dipeptide repeat sequence (EP sequence) [50, 52, 53]. Because of the homology between LAG-3 and CD4, LAG-3 can competitively inhibit CD4 signaling by binding to its classic ligand, major histocompatibility complex (MHC) II. Besides MHC II, several ligands for LAG-3 in different cells and organs have been reported, including α-synuclein fibrils, fibrinogen-like protein 1, TCR-CD3 complex, galectin-3 (GAL-3), and liver and lymph node sinusoidal endothelial cell C-type lectin [54, 55]. When MHC II on APCs binds to LAG-3 on T cells, the KIEELE motif transmits an inhibitory signal to the T cell nucleus, reducing T cell proliferation and Ca^2+^ influx [53] (see Fig. 1). This was proven by Workman *et al*. [56] that depletion of the KIEELE motif leads to the dysfunction of LAG3 in CD4^+^ T cells. The interaction between LAG-3 and the TCR-CD3 complex can occur independently of MHC II and disrupt the co-localization of Lck with CD4 or CD8, thereby inhibiting the activation of both CD4^+^ and CD8^+^ T cells [57, 58]. Consistent with PD-1 and CTLA-4, LAG-3 downregulates T-cell response and causes the immune escape of tumor cells. Clinical research revealed that a higher level of LAG-3 expression is connected to poorer outcomes in malignancies like head and neck squamous cell carcinoma, pancreatic cancer, renal cell carcinoma, etc. [52]. Therefore, LAG-3 is another promising target in immunotherapy.

### 2.4 TIM-3

Monney *et al*. in 2002 [59] reported a cell surface protein specifically expressed on Th1 cells named TIM-3, which is closely linked to T-cell and macrophage activation and the progression of autoimmune diseases [60]. Further studies validated that TIM-3 is also expressed on non-T cells, such as NK cells, myeloid cells, and mast cells. TIM-3 is structured with an extracellular region consisting of an IgV domain, a mucin domain, a transmembrane region, and a cytoplasmic tail that comprises six tyrosines without inhibitory signaling motifs [61, 62]. To date, four ligands of TIM-3 have been reported, including phosphatidylserine (PtdSer), carcinoembryonic antigen-related cell adhesion molecule (CEACAM-1), GAL-9, and high mobility group box 1 (HMGB1) [63, 64, 65]. Clayton *et al*. [66] demonstrated that MHC-TCR binding induces the translocation of TIM-3 from lipid rafts to the immune synapse, where it is ligated to GAL-9 and interacts with CD45 or CD148, subsequently regulating Lck activity (see Fig. 1) [64]. TIM-3 is essential in preventing autoimmune attacks since TIM-3 signaling inhibits Th1 activity and the production of a series of pro-inflammatory cytokines, such as IL-1β and IL-18 [67, 68]. Similar to other immune checkpoints like PD-1 and LAG-3, TIM-3 is abundantly expressed on exhausted T cells. Studies have proved that PD-1^+^/TIM-3^+^ T cells occupy a considerable proportion of tumor-infiltrating lymphocytes (TILs) and anti-TIM-3 treatment can reinstate T-cell proliferation [69]. In some clinical trials, anti-TIM-3 treatment is applied in cancer either alone or combined with other ICIs, and the latter strategy exhibits better anti-tumor efficacy than classic monotherapy [61, 63, 67, 69, 70]. However, the precise role of TIM-3 in immune regulation remains incompletely understood, as some studies indicate that TIM-3 can also function as a costimulatory receptor to enhance immune responses [71, 72]. Moreover, questions still remain about which ligand of TIM-3 mostly affects the anti-tumor capacity of T cells and what pathway the TIM-3 inhibitor mainly blocks.

### 2.5 TIGIT

In 2009, Yu and his colleagues [73] performed genomic screening for costimulatory and inhibitory molecules and discovered a surface protein specifically expressed on T and NK cells, which was soon named TIGIT. Boles *et al*. [74] in the same year also described this protein expressed on follicular CD4^+^ T cells as a ligand for poliovirus receptor (PVR), naming it Washington University Cell Adhesion Molecule (WUCAM). TIGIT has an extracellular IgV domain, a transmembrane region, and an intracellular domain containing an ITIM. TIGIT is induced on T cells for a period after TCR activation and is also observed on regulatory T cells, memory T cells, NK cells, CD8^+^ T cells, B cells, etc. TIGIT is a canonical receptor for PVR, which has the highest affinity among all the receptors of PVR, including CD226 and CD96. TIGIT can competitively inhibit the binding of PVR and CD226, abating the production of proinflammatory cytokines like IL-2 and IFN-γ [73, 75]. The ligation of TIGIT to PVR on the APCs initiates the phosphorylation of PVR and Erk in APCs and promotes the production of immune suppressive cytokines like IL-10 [73]. Also, TIGIT can directly inhibit T cell response by decreasing TCR signaling. Additionally, TIGIT-PVR interaction is reported to inhibit NK cell response, which is dominant over the activating function of CD96/DNAM1-PVR interaction [76]. The upregulation of TIGIT is observed in TILs in a series of malignancies, including non-small cell lung cancer (NSCLC), acute myelogenous leukemia (AML), melanoma, gastric cancer, etc. Several studies showed that anti-TIGIT treatment alone or in combination with anti-PD-1/PD-L1 mAbs can enhance anti-tumor immunity by restoring CD4^+^ T, CD8^+^ T, NK, and T_reg_ cell activity in murine models [52, 77, 78, 79, 80]. Anti-TIGIT agents are currently being assessed in clinical trials and have already presented promising efficacy.

## 3. ICI-Associated Cardiovascular Toxicities

ICIs can cause cardiovascular toxicities by affecting multiple organs and tissues, including myocardium, pericardium, coronary arteries, peripheral vessels, conduction system, coagulation and fibrinolytic system, and endocrine system. In this chapter, we will provide a detailed discussion of each.

### 3.1 ICI Therapy-Related Myocarditis

Myocarditis is a life-threatening irAE, which is generally considered to result from an autoimmune attack on the myocardium by T cells. Normally, a delicate mechanism exists in the cardiac tissue to avoid immune attack. In the presence of ICIs or checkpoint gene deficiencies, myocarditis can develop in mice and cause early death, suggesting that immune-checkpoint signaling builds immune tolerance in the myocardium [30]. In a healthy status, T cells are less present in the myocardium compared to macrophages and accumulate only in pathological conditions. Single-cell transcriptomics detected a significantly increased proportion of activated T cells in the heart tissues from the myocarditis mouse model, which constitute 34% of immune cells in the myocardium, compared to 2% in the control group. Activation markers like *Gzmb*, *Ccl4*, and *Ccl5* were elevated in myocarditis T cells, while naive markers such as *Lef1* and *Ccr7* were higher in T cell controls [81]. Notably, an anti-CD8-depleting antibody, rather than an anti-CD4 antibody, enhances survival in *Pdcd1^-⁣/-^Ctla4^+⁣/-^* mice, highlighting the essential role of CD8+ T cells in ICI-associated myocarditis, contrary to the CD4-dependent myocarditis observed in *Pdcd1^-⁣/-^Lag3^-⁣/-^* mice [81]. This suggests that different ICIs may induce myocarditis through distinct mechanisms [81]. *Ctla4^+⁣/-^ Pdcd1^-⁣/-^* mice model revealed that ICI myocarditis involves an increase in CCR2^+^ macrophages with heightened IFN-γ activity and immune function [30]. This macrophage subset, also found in human ICI myocarditis cases, might take a key role in the disease’s pathology. Their bioinformatic analyses indicate that CD8^+^ T cells may initiate a signaling cascade to CXCL9^+^/CXCL10^+^ macrophages by secreting IFN-γ. A potential feedback loop involving CXCL16/CXCR6 and CXCL9/CXCL10-CXCR3 pathways can facilitate communication between these macrophages and both CD4^+^ and CD8^+^ T cells [30]. This interplay is hypothesized to boost the recruitment and activation of CD8^+^ T cells, possibly leading to cardiomyocyte damage, meriting further exploration.

The impact of the immune checkpoint signaling on the myocardium has been explored in numerous studies. Nishimura *et al*. [4] developed a C57BL/6-PD-1^-⁣/-^ murine model, which exhibited spontaneous glomerulonephritis and arthritis, but myocarditis or cardiomyopathy was not observed in this model. This study also pointed out that PD-1 deficiency, instead of affecting the self-driven proliferation mediated by TCR or IL-2, enhances the proliferative response induced by certain APCs. Lucas *et al*. [82] and Wang *et al*. [83] both established PD-L1^-⁣/-^ MRL mice that can develop myocarditis and pneumonia, and the life span is even shorter among PD-L1^-⁣/-^ MRL-*Fas*^𝑙𝑝𝑟^ mice. In another study by Liu and his colleagues [84], they used Freund’s complete adjuvant (CFA) and a skeletal muscle homogenate from pigs as an immunogen in BALB/c mice, and in the presence of tislelizumab, a PD-1 inhibitor, myocarditis was successfully induced. The preclinical model of ICI-associated myocarditis in *Ctla4^+⁣/-^,Pdcd1^-⁣/-^* mice validated the existence of a gene dosage-dependent genetic and functional interaction between *Ctla4* and *Pdcd1*. Some patients can be predisposed to cardiovascular irAEs due to slight alterations in *Pdcd1*’ and *Ctla4*’s gene dosages [85]. Evidently, PD-1 deficiency presents diverse phenotypes in different animal strains [85]. These results validate that the PD-1 signaling protects the heart tissue from autoimmune attacks, but the specific cardiac antigens targeted by the immune system remain unknown.

Myocarditis can be induced by infectious or non-infectious factors. When viruses kill cardiomyocytes, damage-associated molecular patterns (DAMPs) are released to the interstitial tissue, which recruits DCs and macrophages. Macrophages and DCs present the antigens to T cells in the draining lymph nodes, activating the T cell response in the myocardium [86]. The activated T cells can attack the myocardium when the virus shares a similar antigen with cardiomyocytes or when autoantigens are exposed due to the primary damage [87, 88, 89]. Thus, similar to infection, it’s reasonable to hypothesize that T cells might target myocardium because of the molecular mimicry of tumor antigens and components on cardiomyocytes.

Axelrod and his colleagues performed scTCR-seq on the heart tissues of *Ctla4^+⁣/-^Pdcd^-⁣/-^* mice, and elucidated that α-myosin encoded by *Myh6* can be recognized by TCRs as an MHC-Ⅰ restricted autoantigen to initiate T cell response in myocarditis, and detected α-myosin specific TCRs in ICI-related myocarditis patients [81]. Their study also demonstrated that a large proportion of melanoma tumors with or without ICI treatment expressed *Myh6* [81]. This result is in accordance with prior findings that melanoma patients have a higher risk of developing myocarditis [89]. Interestingly, Wei *et al*. [85] observed no evidence for elevation of serum cytokines or antibodies in *Ctla4^+⁣/-^Pdcd^-⁣/-^* mice, but a higher level of serum troponin, which suggested cardiac tissue damage. Myers *et al*. [90] analyzed endomyocardial biopsy from myocarditis and dilated cardiomyopathy patients, and proved that human cardiac myosin (HCM) peptides S2-16 and S2-28 can be recognized as endogenous toll-like receptor 2 (TLR2) ligands by CD14^+^ monocytes. After the stimulation, CD14^+^ monocytes produce IL-6 and TGF-β, facilitating Th17-cytokine secretion such as IL-17A, which participates in the progression of myocarditis [90].

A number of research findings point to an intriguing perspective that mitochondria might be targeted in the development of myocarditis. In Liu *et al*.’s study [84], upregulated levels of Fas, FasL, LC3, p62, and anti-mitochondrial antibody-M2 (AMA-M2) are detected in myocarditis mice. Fas/FasL, LC3, and p62 are related to apoptosis and autophagy, respectively, and AMA-M2 is found in various autoimmune diseases. Likewise, proteomic analysis revealed that in ICI-induced myocarditis, the expressions of mitochondria-related molecules mitofusin (MFN)2, glycogen synthase kinase (GSK)3β, mechanistic target of rapamycin kinase (mTOR), and protein tyrosine phosphatase non-receptor type 11 (PTPN11) are elevated [91]. Schulze *et al*. [92] induced myocarditis in murine models with Coxsackie B3 virus and detected anti-adenine nucleotide translocator (ANT) antibodies in the sera, which caused impaired ATP translocation and cardiac dysfunction. Cardiomyocytes are cells with extremely high metabolic activity and possess a large amount of mitochondria. Once the mitochondria are targeted and impaired, ROS is generated and exacerbates inflammatory responses. It’s been confirmed that myocardial infarction is more likely to occur in males, with a ratio of 3.5:1 male to female reported in a recent study [93]. It is thought that the protective effect of estrogen in reducing cardiovascular disease before the menopause is mediated by ERα expression in mitochondria [94]. Taken together, mitochondria may contribute to the development of myocarditis through several mechanisms, including providing antigens as targets; causing metabolic dysfunction following damage, which leads to cardiac dysfunction; and releasing ROS that exacerbate inflammatory responses in myocardial tissue. However, further research is still needed to determine whether myocardial mitochondria are the initial cause of ICI-induced myocarditis.

Currently, no reliable theory exists to explain which type of ICIs is more likely to induce myocarditis. However, a large-scale clinical study has demonstrated that combination therapy of anti-PD-(L)1 and anti-CTLA4 is more likely to induce myocarditis than monotherapy, with an incidence rate of 1.33% [11]. And the incidence rates in anti-PD-1 or anti-PD-L1, and anti-CTLA4 monotherapy are 0.41% and 0.07%, respectively. The dual therapy of anti-LAG3 and anti-PD-1 also increased the incidence to 1.7%, versus 0.4% in anti-PD-1 alone [95]. These findings suggest that the combination of ICIs not only enhances antitumor immune responses but also exacerbates autoimmune reactions in the body.

**Fig. 2.**
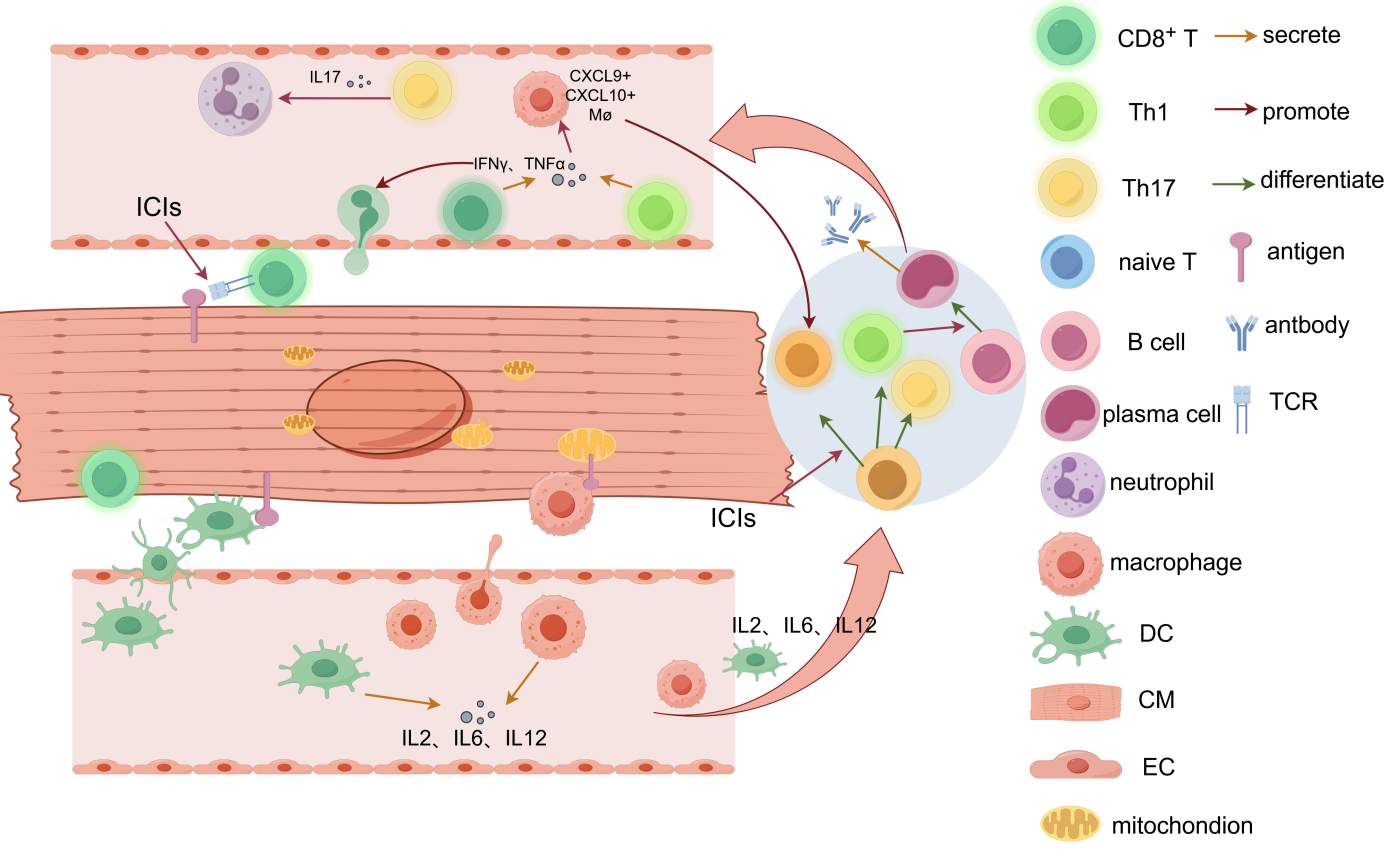
**Mechanism of ICI-induced myocarditis**. Cardiac auto-antigens can be recognized and presented to naive T cells in the draining lymph nodes by APCs such as macrophages and DCs. Meanwhile, APCs secrete pro-inflammatory cytokines, such as IL-2, IL-6, and IL-12. In normal status, this interaction will not start an immune reaction due to the immune tolerance maintained by immune checkpoints. When ICIs are applied, activated CD4^+^ (Th1, Th17) and CD8^+^ T cells are generated. Activated CD8^+^ T cells target cardiac antigens and directly lead to T-cell infiltration in the myocardium. Th1 and activated CD8^+^ T cells both secrete IFN-γ and TNF-α, which, on the one hand, promote T cell infiltration, and on the other hand, initiate a signaling cascade to CXCL9^+^/CXCL10^+^ macrophages. The CXCL9^+^/CXCL10^+^ macrophages recruit CD8^+^ T cells as a positive feedback loop. Th1 cells also initiate B-cell activity and plasma cell differentiation, causing Ig deposition around cardiomyocytes. Th17 cells promote the recruitment of neutrophils by producing IL-17. Mø, macrophage; Th, helper T cell; CM, cardiomyocyte; EC, endothelial cell; IFN-γ, interferon-γ; TNF, tumor necrosis factor; IL, interleukin. This figure was created by figdraw (https://www.figdraw.com/#/).

In summary, while the exact mechanism of ICI-related myocarditis and cardiomyopathy remains unclear, existing studies support a plausible assumption. Potential antigens exist in the myocardium, such as α-myosin, cardiac troponin I (cTnI), ANT, among others. Regardless of the presence of tumors, this TCR signaling can initiate an immune response against the myocardium. But in normal status, due to the immune tolerance maintained by inhibitory checkpoint signaling, cardiomyocytes are free of attack by specific immunity, and few T cells are resident in the myocardium. Deficiency in checkpoint-related genes or the presence of ICIs disrupts immune tolerance, unlocking autoimmunity against myocardial antigens, which leads to the accumulation of particularly CD8^+^ T cells in the myocardium (see Fig. 2). In the presence of tumors, especially when tumor cells express similar molecules to cardiomyocytes, the autoimmune response in heart tissue might be more vigorous [89].

### 3.2 ICI Therapy-Related Pericardial Diseases

Pericardial diseases, pericarditis, for instance, exhibit a lower incidence compared to myocardial diseases. In a large prospective study conducted by Gong *et al*. [96], the patients treated with ICI presented an incidence of 1.57 events per 100 person-years and a seven times increase in the risk of developing pericardial diseases compared to the untreated group. In an observational study, Salem *et al*. [11] reported a rate of 0.36% of anti-PD-1/PD-L1 monotherapy-related pericardial diseases in all individual case safety reports (ICRS) reported with ICIs. However, anti-CTLA-4 agents were reported to be unrelated to the risk of pericardial toxicities [97]. Unlike myocarditis, the lack of generally accepted animal models and clinical research hinders the exploration of the mechanism in the development of PD-1 inhibitor-related pericardial diseases.

Altan *et al*. [98] reported three cases of pericarditis in which they observed a large accumulation of CD4^+^ and CD8^+^ T cells around the same share, as well as a certain amount of CD68^+^ macrophages beneath the fibrinous layer on the biopsy. Occasional reactive mesothelial cells and CD20^+^ B cells are also observed [16]. Another case report showed predominant infiltration of CD4^+^ T cells, expressing CD4^+^ and FOXP3^+^ [99]. These findings are different from the pathology results observed on myocarditis biopsies, suggesting that the pericardial tissue possesses a relatively specific immune microenvironment. Besides, in some cases, malignant cells are detected in the pericardial effusion, which makes it difficult to validate the cause of pericarditis [100]. Moreover, radiation therapy combined with ICIs is thought to be more likely to cause pericardial diseases [16]. Pericardial diseases are more commonly diagnosed in cases of lung cancer, which also supports the argument mentioned above, since radiotherapy is more frequently applied to lung cancer. A hypothesis for this increased risk of pericarditis is that radiotherapy induces localized tissue necrosis, which causes the release of DAMPs. DAMPs can activate the innate immunity, such as macrophages, creating a pro-inflammatory environment, where T cells are randomly activated. These antigens activate T cells, leading to an attack on the pericardium. A possibility exists that tumor invasion into the pericardium leads to inflammation [16]. Additionally, pericarditis that happened after ICI treatment might be related to primary or recurrent infection. Chu *et al*. [101] reported a case in which, after anti-PD-1 therapy, the NSCLC patient developed a pericardial tamponade due to a hypersensitive response to tuberculosis (TB) reactivation. Interestingly, they found that PD-1 signaling in the two diseases does not co-promote each other to evade the host immunity. When TB was controlled, the pericardial effusion was PD-L1 negative but the expression was upregulated in cancer cells due to tumor progression.

In brief, although initiating antigens and underlying mechanisms for pericardial diseases are far from clear, it’s agreed that pericardial diseases are developed under the influence of multiple factors. With the presence of ICIs, T-cell response can be induced when identical antigens on the pericardium and tumor cells are targeted by TCRs. Combined therapy with radiation can be a risk factor. PD-1 inhibitors might also enhance the immune reaction initiated by infection or induced by autoimmune disorders. Future research requires the development of animal models capable of reliably simulating the pathological features of human ICI-related pericarditis, which is fundamental to advancing mechanistic studies. Moreover, multi-omics studies analyzing patients’ sera, pericardial effusions, and pericardial tissues will establish immune cell atlases and describe the molecular characteristics during pericarditis, which help uncover crucial subclusters, pathways and biomarkers.

### 3.3 ICI Therapy-Related Arrhythmias

Arrhythmia and conduction diseases are common cardiovascular toxicities of ICI therapies. The incidence of ICI-induced arrhythmia is reported to be around 1.5% [102]. Male and aged patients (≥65 years old) are more likely to develop ICI-associated arrhythmias. These cases are more commonly reported in lung, thymus, and pleura cancer patients, especially in metastatic NSCLC or melanoma patients, and the type of arrhythmias can range from tachycardia, atrial or ventricular fibrillation, to bradycardia, sick sinus syndrome, atrioventricular block, and cardiac arrest [103, 104]. Importantly, studies indicate that anti-PD-1/PD-L1 therapies exhibited substantially increased reporting odds ratios concerning arrhythmia, while anti-CTLA-4 therapy is considered to be unassociated with the incidence [103, 105]. Meanwhile, dual therapy poses a higher risk of arrhythmias compared to monotherapy [105]. ICI-associated arrhythmias can be newly emerged or pre-existing, and primary or secondary to concurrent cardiovascular toxicities, such as myocarditis, cardiomyopathies, cardiac failure, and coronary diseases [5]. Due to the relatively low mortality rate, the pathophysiological mechanisms underlying ICI-induced arrhythmias have not been fully investigated.

Arrhythmia is a disorder of cardiac rhythm triggered by abnormal impulse formation, conduction, or both [106]. Therefore, studying the etiology and pathology of arrhythmias during immunotherapy can be quite different from other adverse events, as arrhythmias can be induced by various factors like drug toxicities, primary cardiac diseases, nervous or endocrine disorders, electrolyte disturbances, and even tumor invasion. From the perspective of ICI, the occurrence of immune-related arrhythmias can be considered a direct or indirect effect of immunotherapy.

ICIs induce the activation and monoclonal proliferation of effector T cells. Similar to immune myocarditis and pericarditis, in the presence of cross-reactive antigens, ICIs may trigger T-cell attacks on the sinoatrial node, atrioventricular node, and conduction bundle cells, which is considered the direct effect of ICIs. It’s been repeatedly reported that arrhythmias can occur in patients with ICI-induced myocarditis [13, 107, 108]. The development of arrhythmias may result from T-cell infiltration in the myocardium, especially in the nodal area and conduction system [13]. In two case reports in which myocarditis with complete heart block occurred after nivolumab therapy in metastatic melanoma patients, the cardiac autopsies presented similar infiltration of CD3^+^ T cells and CD68^+^ macrophages in the cardiac sinus and conduction system, and plentiful CD4^+^ and CD8^+^ cells were observed [108]. Surprisingly, two studies confirmed that in AF patients, the expression of PD-1 on CD4^+^ T cells is decreased compared to controls, while remaining in CD8^+^ T cells, which suggests that CD4^+^ T cells might be more critical for the occurrence of atrial fibrillation (AF) [109, 110].

The indirect effects of ICI on cardiac conduction include increased ROS formation, pro-inflammatory factor release, and exacerbated myocardial remodeling. In a study by Fu *et al*. [111], PD-1-deficient mice exhibited significantly shortened effective refractory periods (ERPs) at each atrial site and enhanced dispersion. Increased ROS generation and immune factors such as IL-17, TNF, and IFN-γ are detected, and accumulated collagen fibers are observed in atrial tissue. These results suggested that checkpoint deficiencies can induce atrial remodeling by ROS generation, inflammation, and cardiac fibrosis, which promotes the formation of reentrant circuits, thus raising the risk of AF Besides, pro-inflammatory factors, such as IFN-γ, IL-2, IL-6, and IL-10, are significantly increased in the peripheral blood of AF patients, while IL-17A and TNF remain normal. Meanwhile, CD69 and HLA-DR are upregulated on CD3^+^ T cells in the AF group, suggesting enhanced activation of T cells, while PD-L1 is downregulated on myeloid DCs (mDCs) [109]. ICIs enhanced the mDCs’ ability to initiate T cell response and secretion of pro-inflammatory cytokines, aggravating the inflammation in the myocardium, which affects the conduction system.

It’s well-known that the endocrine system is essential in the regulation of cardiac rhythm. As some cases have reported that ICI therapy can disturb the endocrine system, arrhythmia may be developed due to endocrine disorders. Various endocrine organs, including the thyroid, pituitary, and adrenal gland, are affected by ICIs [13, 112]. Hyperthyroidism and hypocortisolism induced by ICIs can interrupt cardiac rhythm. For instance, Guo *et al*. [113] reported a case in which coronary artery spasm and ventricular tachycardia developed due to hyperthyroidism induced by a PD-1 inhibitor.

**Fig. 3.**
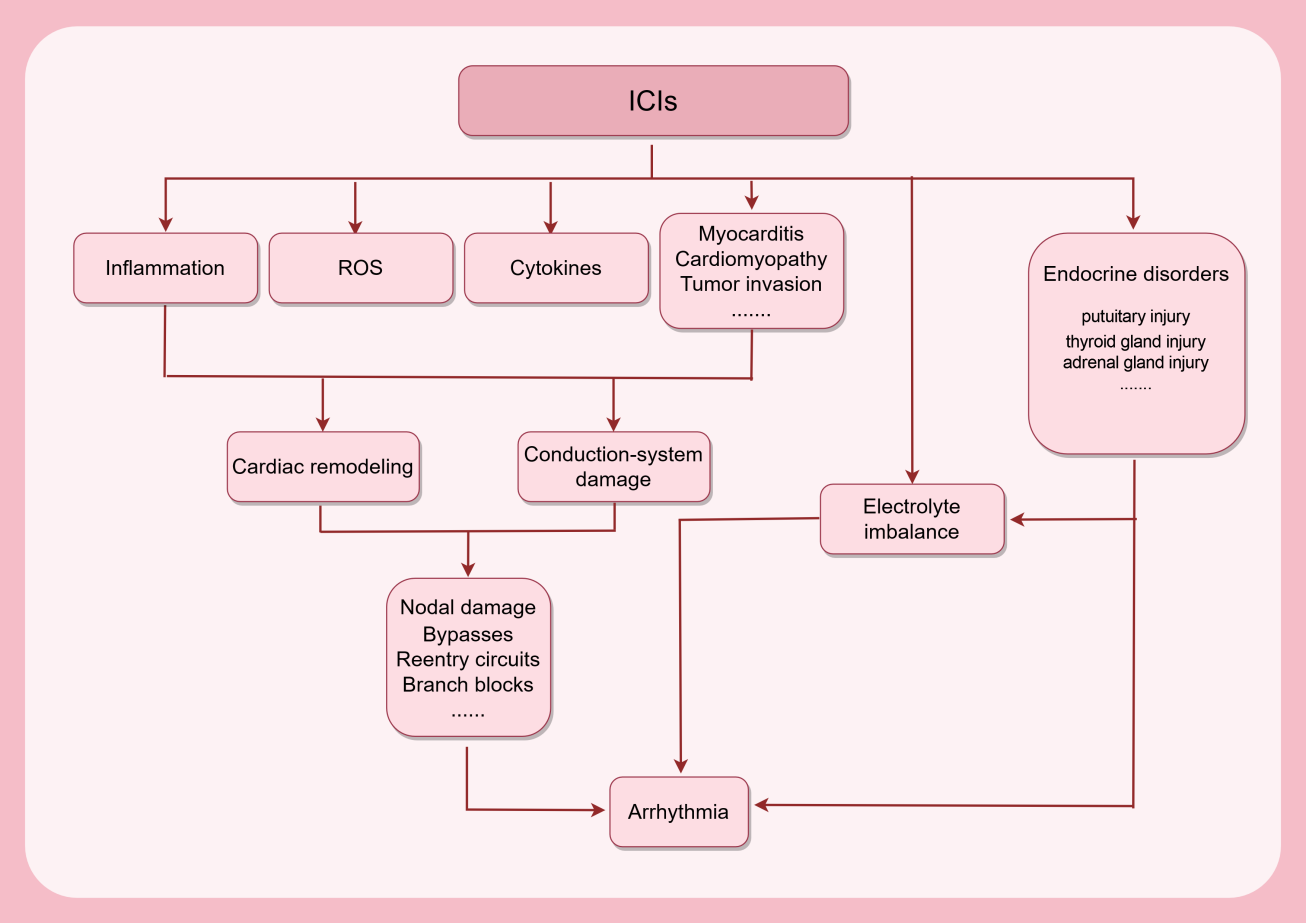
**Development of ICI-related arrhythmias**. The use of ICIs can trigger an immune response in the myocardium, increasing ROS production and (non)inflammatory cytokine secretion, which will cause cardiac remodeling with or without myocarditis and cardiomyopathy. Immune cell infiltration will bring damage to the conduction system. Consequently, a series of structural alterations might occur in the conduction system. ICI treatment can disturb the electrolyte balance, perturbing the automaticity of rhythmic cells. Meanwhile, ICIs can cause injuries to endocrine organs, which directly or indirectly contribute to the progression of arrhythmias. ROS, reactive oxidative species. This figure was created by figdraw (https://www.figdraw.com/#/).

We summarized the potential mechanisms of ICI-induced arrhythmias in Fig. 3. Overall, ICIs induce arrhythmias through multiple ways. First, arrhythmia can be secondary to myocarditis and cardiomyopathies. Second, ICIs can not only trigger T cell attack on conductive cells, but also promote ROS generation and non-inflammatory cytokine release, both contributing to the cardiac remodelling and conduction system damage. Subsequently, bypasses, reentry circuits, and branch blocks may form in the conduction system. In addition, systemic impacts, particularly endocrine disruption resulting from ICIs, are also influential during the progression of arrhythmias.

### 3.4 ICI Therapy-Related Heart Failures

**Fig. 4.**
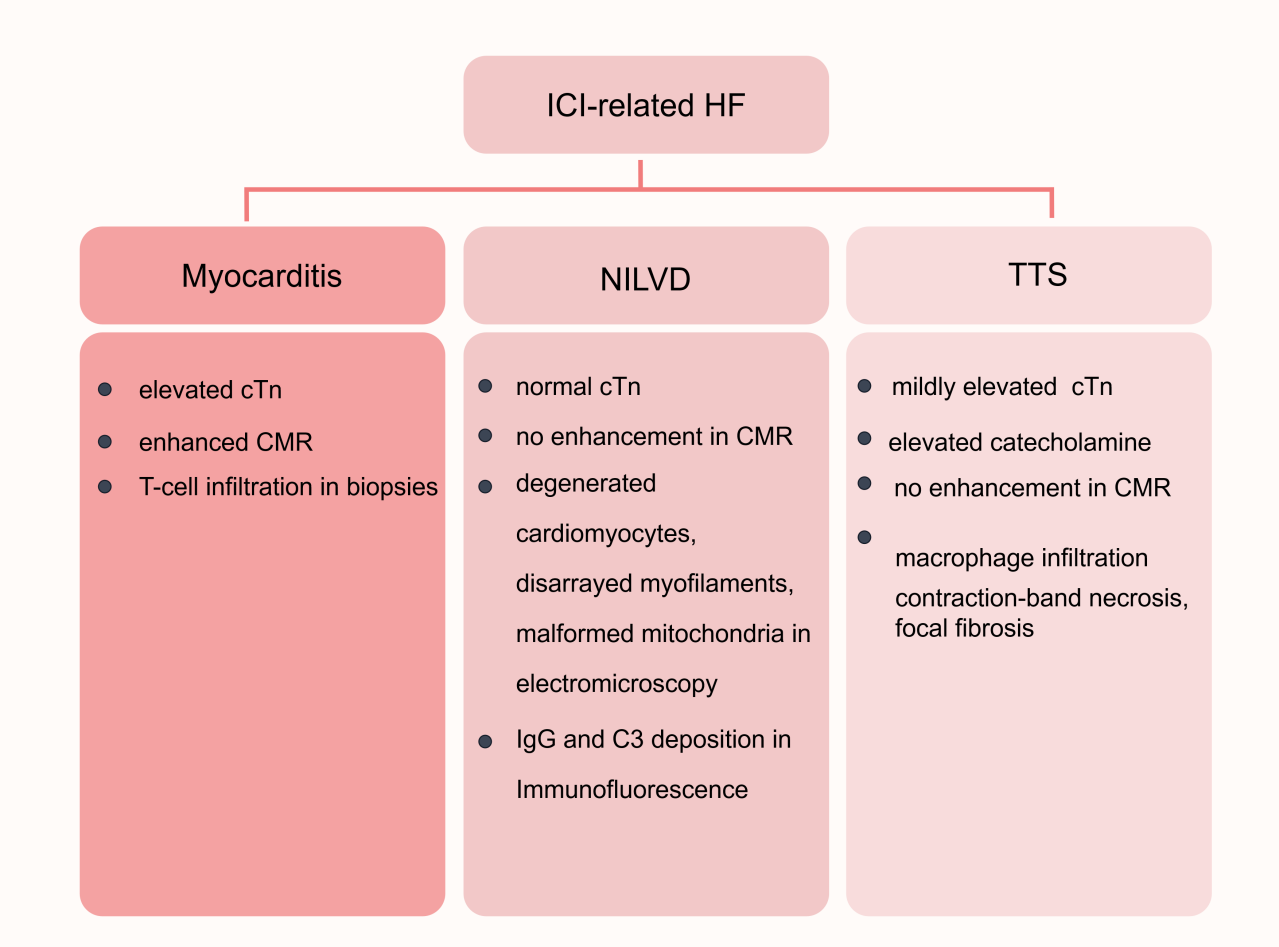
**Classifications of ICI-related HF**. ICI-related HFs include myocarditis-associated HF, NILVD, and TTS. ICI-related myocarditis exhibits an elevated cTn level and enhancement on CMR and features T-cell infiltration in biopsies. In NILVD, the cTn level is typically normal, and no enhancement is observed on CMR. NILVD is characterized by degenerative and disorganized cardiomyocytes, with deformed mitochondria and IgG and C3 deposition. TTS is usually accompanied by a significantly elevated catecholamine and mildly elevated cTn level, with no enhancement on CMR. Macrophage infiltration, contraction-ban necrosis, and focal fibrosis can be observed in the biopsies of TTS. HF, heart failure; NILVD, non-inflammatory left ventricular dysfunction; TTS, Takotsubo syndrome; cTn, cardiac troponin; CMR, cardiac magnetic resonance; IgG, immunoglobulin G; C3, complement 3. This figure was created by figdraw (https://www.figdraw.com/#/).

Heart failures can occur following myocarditis and cardiomyopathies, but in some cases, ICIs can cause ventricular dysfunction and heart failures in the absence of myocarditis (see Fig. 4). In a descriptive analysis by Escudier and his colleagues [114], left ventricular systolic dysfunction was observed in 79% of the enrolled cases of ICI-related cardiovascular toxicity, which is higher than the proportion presented with elevated troponin, myocardial edema or late MRI enhancement, suggesting a subset of heart failure. This subset, unaccompanied by myocarditis, is considered functional and non-inflammatory, which features an absence of elevated troponin, imaging evidence of myocardial inflammation, and immune cell infiltration on biopsies [5, 115, 116]. Non-inflammatory left ventricular dysfunction (NILVD) exhibits a longer median time to presentation than myocarditis and can be accompanied by right ventricular dysfunction as well [116]. The recognition of NILVD suggests the existence of a non-inflammatory mechanism leading to cardiac dysfunction.

In the study by Nishimura *et al*. [117], PD-1^-⁣/-^ BALB/c mice presented fatal dilated cardiomyopathy, and histological examination showed no evident infiltration of mononuclear cells, while electron microscopy exhibited degeneration of cardiomyocytes, disarrayed myofilaments, and malformed mitochondria. Immunofluorescence showed immunoglobulin G (IgG) and complement 3 (C3) deposition around cardiomyocytes in affected hearts, and high-titer IgG reactivity to a 33-KDa protein selectively expressed on the surface of cardiomyocytes was detected in the sera from affected animals [117]. This result indicates a humoral immune approach to PD-1 inhibitor-related dilated cardiomyopathy (DCM). Besides, Okazaki *et al*. [118] also employed BALB/c-PD-1^-⁣/-^ DCM mice and found high-titer autoantibodies targeting a cardiac-specific 30-KDa protein, which is proven to be cTnI. They proposed that a certain amount of cTnI can be expressed on the membrane of cardiomyocytes and be recognized by cTnI-specific antibodies, which increase Ca^2+^ flux on the L-type Ca^2+^ channel and lead to cardiac dysfunction and DCM [118]. Consistent with PD-1 deficiency, PD-1 inhibitors was also reported to decrease cardiac function and induce senescence in C57/B16 mice. Xia and his colleagues [119] demonstrated that a cellular senescence-related microRNA, miR-34a-5p, transferred by exosomes, is upregulated in macrophages pretreated by PD-1 inhibitor, which propels cardiac aging by aiming serine/threonine-protein phosphatase 1 regulatory subunit 10 (PNUTS). Xia and his team [120] also proposed that PD-1 inhibitors can promote the differentiation of M1 macrophages by regulating the miR-34a/KLF4 pathway to cause cardiac injury. Therefore, it can be concluded that humoral immune reactions against molecules on cardiomyocytes can be initiated in the presence of ICIs. Moreover, ICIs can affect macrophage differentiation and consequently lead to cardiac aging and ventricular dysfunction.

Takotsubo syndrome (TTS) is another cardiovascular adverse event of ICI therapy, which features acute, transient regional left ventricular dysfunction with coronary atherosclerosis [121]. Stress, increased catecholamine production, microvascular dysfunction, and multivessel coronary spasm are possible mechanisms that lead to Takotsubo cardiomyopathy [122, 123]. Aborted myocardial infarction with spontaneous recanalization, acute obstruction of the left ventricular outflow tract, and stunning of myocardium mediated by catecholamine are also hypotheses on the initiation of TTS [124]. Commonly, contraction band necrosis is the histological characteristic [124, 125]. It’s been reported that emotional stress-induced left ventricular dysfunction exhibits remarkably elevated catecholamine levels. Infiltration of macrophages and mononuclear lymphocytes and necrosis of contraction bands are observed in the biopsies [125]. These features are similar to those found in ICI-induced TTS [126, 127, 128]. As shown in the endomyocardial biopsy, malignant cells and lymphocyte infiltration are absent, and focal fibrosis and few CD68^+^ macrophages are observed [121]. Hence, it can be inferred that ICI-induced TTS possibly results from excessive catecholamine released by the impaired adrenal gland or coronary vasospasm due to the interaction between vascular walls and ICIs.

### 3.5 ICI Therapy-Related Vasculitis

Vasculitis contains a series of diverse autoimmune diseases that can attack different-sized vessels thereby causing damage, including inflammation-induced centripetal intimal hyperplasia of blood vessels, aortic wall thinning, and aneurysm formation. The vascular wall possesses a distinct barrier structure that protects it from autoimmune attacks. ICIs can cause different types of vasculitides, such as giant cell arteritis (GCA), aortic arteritis, anti-neutrophil cytoplasmic antibody (ANCA)-associated vasculitides (AAVs), neurologic primary vasculitis [129], leukocyte-crushing vasculitis (LCV) [130], cryoglobulinemic vasculitis, and drug-induced vasculitis [131].

GCA, the most common type of vasculitis associated with ICI therapies, is an autoimmune vasculitis focused on the aorta and its medium and large branch vessels being affected, marked by a substantial infiltration of effector T cells [132, 133], which may lead to blindness and stroke as the disease progresses [134]. In the vessel wall, DCs, multinucleated giant cells (MGCs), macrophages, and CD4^+^ T cells form a non-necrotizing granuloma that penetrates from the peritoneum to the middle layer and destroys the elastic lamina, damaging the vascular wall and causing injuries such as elastic lamina fragmentation, luminal stenosis and occlusion, vessel wall entrapment, and aneurysm formation [132, 135]. IL-9, IL-21, IL-17, and IFN-γ are produced by CD4^+^ T cells, promoting neoangiogenesis and hyperplasia in the intima [136]. During active disease, macrophages release IL-6, IL-1β, and TGF-β. Myofibroblast proliferation and neoangiogenesis are supported by macrophage growth factor (MGF) [137, 138]. MGCs were reported to be present in nearly half of the patients, and were found to be concentrated in the medial layer [139], distinguishing them from Takayasu arteritis. Watanabe *et al*. [132] found that PD-1^+^ T-cell accumulation in lesions was directly proportional to the severity of vasculitis. Increased production of pro-inflammatory cytokines is related to a higher density of PD-1^+^ T cells in the intima, while thicker intimal layers are linked to a higher microvascular density [132]. In one study, myofibroblasts expressing α-smooth muscle actin (α-SMC) in the proliferative intima were two-three times thicker in the PD-1 inhibitor-treated group than in the control group [140].

The progression of GCA is itself influenced by a variety of pathways, including the JAK/STAT signaling pathway, the PD-1/PD-L1 pathway, the CD28 pathway, the NOTCH1-Jagged1 pathway, etc. [135, 141]. Vascular DCs and CD4^+^ T cells are crucial factors in the progression of GCA. In GCA, vascular DCs reside in medium- and large-sized vessels, and express a high level of the costimulatory ligand CD80/CD86 [11] and a low level of the co-inhibitory ligand PD-L1 [132]. Vascular DCs present vascular pathogenic antigens from the vessel wall, exposing the vessels to auto-immune attacks [142, 143]. The imbalance between powerful co-stimulation and ineffective co-inhibition leads to the clonal expansion of T cells [135]. Defects in co-inhibitory pathways are hallmarks of GCA [140]. Abnormal expressed NOTCH1 receptors and dependence on unantagonized co-stimulatory signaling are characteristics of GCA-related CD4^+^ T cells. A variety of complex pathogenic cascades culminate in vessel wall disruption and intimal hyperplasia [141].

Under normal physiological conditions, the PD-1/PD-L1 signaling serves to provide negative signaling to block T-cell activation and expansion and prevent inflammation-related tissue destruction. As opposed to a high PD-L1 expression in arteries of healthy populations that contributes to immune privilege, the absence of PD-L1 in DCs in GCA results in unopposed T-cell activation signaling in patients. This protective mechanism is disrupted in GCA. Interestingly, pathways and proteins related to CTLA-4 are upregulated in the blood and aortic tissues of GCA patients. Despite the decreased amount and diminished activation/suppression of T_regs_ in GCA patients, CTLA-4 is still particularly upregulated [133]. This probably explains why CTLA-4 inhibitors were linked to a higher incidence of GCA compared to PD-(L)1 inhibitors, despite the latter’s more prevalent usage and irAEs documentation. As PD-1 signaling is inhibited in vascular DCs [140], naive CD4 T cells fail to convert to T_regs_ [144], leading to uncontrolled and hyperactivated lesion T cells that react to stimulation normally inadequate to initiate T-cell reaction. Moreover, Th1 and Th17 cells are enriched [145] and positively correlate with arterial tissue injury [146], and the memory T cells make the lesion self-sustaining [135]. In a permissive tissue environment created by defective PD1/PD-L1 immune checkpoints, PD-1^+^/CD4^+^ T cells enter the immune-privileged vessel wall and differentiate into multiple classes of differentiated effector T cells that secret cytokines (IL-17, IL-21 and IFN-γ) that orchestrate the destruction of the vascular wall and drive an inflammatory response of endothelial activation, intimal hyperplasia, intramural neoangiogenesis, and luminal stenosis [132, 141]. PD-L1 expression was decreased on GCA macrophages and DCs, whereas PD-L2 expression was unaffected, but PD-L2 could not replace the lack of PD-L1-dependent signaling [147, 148].

In one study, normal human arteries were grafted into immunocompromised mice, and mononuclear cells in the peripheral blood from GCA patients were then transplanted to reconstitute chimeric mice, and vasculitis developed within 1-2 weeks. PD-1 inhibitor led to a strong upregulation in IL-1β, IL-6, TNF-α, IL-7, IL-15, and DCs involved in granulomatous lesions in chimeras, and a substantial amount of different T-cell lineages were recruited to the affected vessel intima. LPS and IFN-γ can induce PD-L1 expression on DCs, and it was found that GCA was attenuated in response to both stimuli [149].

Macrophage aggregation in the vessel wall is not unique to GCA, and overexpression of MMP-9-producing macrophages is also present in other granulomatous diseases, including granulomatous polyangiitis arteriosa (GPA) [150]. GPA is an autoimmune small-vessel vasculitis usually connected to granuloma-caused tissue damage. Neutrophilic extracellular traps (NETs), composed of neutrophils, are thought to act as inflammatory foci. Correspondingly, small and medium-sized vessels can be affected by anti-neutrophil cytoplasmic antibody-associated vasculitis (AAV). Mixed monocytes and neutrophils comprise vascular lesions at the early stage of AAV, which are gradually replaced by monocyte- and macrophage-dominated inflammation during progression [151]. A lower amount of PD-L1^+^ monocytes was observed in AAV patients with PR3 or MPO-ANCA^+^ than normal group, which may be associated with a low expression of the crucial domain in CKLF-like Marvel transmembrane structural domain 6 (CMTM6) [152].

AAVs are another group of vasculitides that can be induced by ICIs, which involve severe, systemic small-vessel vasculitis. They are characterized by the development of autoantibodies to neutrophil proteins, such as PR3-ANCA (anti-proteinase 3) or myeloperoxidase (MPO-ANCA) [153]. The onset of ANCA-associated vasculitis is associated with multiple factors, including infections, drugs, and genetic susceptibility. Many pathogens, such as Streptococcus, can produce pyrogenic toxins, simultaneously activating both autoreactive B and T cells [154]. Drugs like pyrimethamine, minocycline, and isoniazid etc. are related to the development of AAVs [155]. A clinical study demonstrated that HLA-DRB1*09:01, commonly found in East Asian populations, is strongly associated with microscopic polyangiitis (MPA) [156]. From these results, we can infer that whether ICIs induce vasculitis is highly correlated with the characteristics of the users. Unlike other cardiovascular toxicities, B cells play a crucial role in the development of AAVs. B cells not only participate in AAV pathogenesis as precursors to ANCA-producing plasma cells, but also present antigens to T cells to stimulate T cell activation. They secrete pro-inflammatory factors such as IL-6 and TNF, suppressing the anti-inflammatory activity of regulatory T cells while promoting the differentiation of effector T cells. Disruption of T cell immune homeostasis is important in both the initiation and subsequent progression of AAVs. TGF-β and IL-6 produced by dendritic cells can induce naive T cells to differentiate into Th17. Dendritic cell-derived IL-27 stimulates Th17 cells to produce IL-17, which in turn induces macrophages to release proinflammatory mediators that pre-activate neutrophils [157]. Thus, we can infer that ICIs exacerbate the activation of T and B cells. Activated helper T cells, particularly Th17 cells, will secrete increased amounts of IL-17, ultimately inducing neutrophil activation and the formation of AAVs.

LCV is a group of diseases that features neutrophilic infiltration primarily affecting the skin in a vasculitis pattern. The specific pathogenesis of LCV remains unclear, but it is generally believed to be associated with the deposition of circulating antigen-antibody complexes in blood vessels, which activates the complement system and subsequently recruits neutrophils [158]. Therefore, the pathogenesis of ICI-induced LCV likely resembles that of AAVs, wherein activated B cells and plasma cells promote increased complex deposition, while helper T cells enhance neutrophil activity.

Together, the mechanisms by which ICI induces different types of vasculitis share commonalities as well as differences. In terms of differences, in ICI-induced GCA, macrophages play a crucial role both in secreting inflammatory mediators that damage blood vessels and in forming giant cells that penetrate the vascular wall. In AAVs or LCVs, more active neutrophils are clearly a key factor in disease progression. However, T cell activation plays a crucial role in all these processes. The occurrence of ICI-induced vasculitis is associated with multiple factors, including the antitumor strategies employed, drugs, living environments, chronic infections, genetic susceptibility.

### 3.6 ICI Therapy-Related Atherosclerosis

Atherosclerosis (AS) is a chronic inflammatory disease primarily affecting the medium to large arteries, which serves as the pathological basis of a range of cardiovascular diseases. The process is initiated by lipid metabolic disorders and characterized by lipid deposition and inflammatory cell accumulation in vascular walls. The subsequent fibrosis, calcification, and even ruptures can ultimately lead to stenosis, thrombosis, and hemorrhage, potentially limiting survival in post-ICI therapy patients.

Recent studies using scRNA-seq have shown that in human atherosclerotic plaques, CD8^+^ T cells are significantly enriched, comprising about 65% of plaque immune cells. This enrichment contrasts with the distribution of CD4^+^ T cells, which are more prevalent in circulation, emphasizing the role of cytotoxic T cells in AS [159, 160]. Cytokines secreted by Th1 cells, including TNF-α, IL-18, IL-12, and IFN-γ, are able to interact with macrophages, regulate macrophage conversion to M1 subtype, and promote inflammatory responses [161]. On the contrary, the concomitant decrease in the M2 subset results in an anti-inflammatory effect, consistent with reduction in cytokines such as TGF-β and IL-10. The PD-1 pathway is important in the progression of AS. As mentioned previously, PD-1 inhibitors may facilitate the polarization of M1 macrophages [120]. In addition, TNF-α and IFN-γ upregulate the PD-L1 expression on myocardial endothelial cells, leading to exacerbation of myocarditis and a pro-inflammatory environment [162]. In vasculitis involving major vessels, low expression of PD-L1 disrupts checkpoints and allows unrestricted T-cell reaction. In contrast, atherosclerotic plaques exhibit a high level of PD-L1 [159], suggesting that PD-1/PD-L1 inhibition may exacerbate inflammation within plaques.

In PD-1/PD-L1-knockout murine models of AS, it was observed that PD-1/PD-L1 defects increased the number of CD4^+^ and CD8^+^ T cells and pro-inflammatory cytokines such as TNF-α and INF-γ, resulting in a higher atherosclerotic burden in the aorta [163, 164]. Thus, it may be possible to alleviate inflammation and ameliorate the progression of AS through upregulating PD-1/PD-L1 expression.

The balance between the expression of positive and negative ligands and receptors on APCs and T cells jointly regulates the activation of naïve T cells [165]. DCs, as the most important APCs, are the most potent inducers of T-cell response and are significantly elevated in AS [166]. On one hand, high expression of MHCs in DCs induces the conversion of macrophages to the M1 phenotype upon binding with DCs and T cells, increasing pro-inflammatory cytokines, oxidative stress, and inflammation, thereby destabilizing atherosclerotic plaques [167]. On the other hand, DCs stimulate immune responses by binding to T cells and presenting oxLDL-derived antigens in atherosclerotic plaques [168]. Lee *et al*. [165] found that in CAD patients, PD-1 and PD-L1 were downregulated in T cells and mDCs, respectively. The PD-1 and TIM-3 co-expression is mainly upregulated in circulating atherosclerotic CD8^+^ T cells, and Qiu *et al*. [169] found that anti-PD-1 or anti-TIM-3 treatments exacerbated the predominance of Th1-driven pro-inflammatory responses. However, a recent study by Fan *et al*. [170] drew an opposite conclusion: they found that PD-1 inhibitors significantly reduced plaque size in AS patients. Using scRNA-seq, they identified a distinct LMNA^+^/PDCD1^+^ T cell cluster in AS plaques, which is in an activated state instead of an exhausted state like PD-1^+^ TILs. FcγR-expressing myeloid cells can capture the Fc-binding PD-1 inhibitor, which acts as a substitute for PD-L1, inhibiting the activation of PD-1^+^ T cells, thus exerting an anti-AS effect [170]. This study not only suggests a novel therapeutic strategy for treating AS but also raises a question about the existence of the same PDCD^+^ activated T cells in other inflammatory diseases. However, as the authors acknowledge in the paper, the study still has limitations. Due to ethical constraints, the anti-atherosclerotic effects of PD-1 antibodies have not been demonstrated in non-tumor populations. Additionally, single-cell sequencing studies tend to provide a snapshot of a disease at a specific point in time, rather than accurately depicting its progression. Therefore, as most studies indicate, the prevailing view remains that the use of ICIs is more likely to promote the progression of atherosclerosis than to mitigate it.

The CTLA-4 pathway also plays an important role in the formation of plaques. In a study utilizing the apolipoprotein E-deficient (*Apoe*^-⁣/-^) mouse model, Matsumoto *et al*. [171] demonstrated that overexpression of CTLA-4 significantly reduced the accumulation of CD4^+^ T cells and macrophages at the aortic root and attenuated the growth of atherosclerotic lesions. This may be attributed to the less proliferating and secreting CD11c^+^ DCs on which the expression of CD80 is downregulated, and the overall suppressed T cell proliferation by inhibited co-stimulatory pathway [171]. Given the evidence supporting CTLA-4’s roles in mitigating AS, it is plausible to assume that CTLA-4 inhibitors could potentially initiate or exacerbate AS. A study by Poels *et al*. [172] explored the impact of ICIs on macrophage-driven vascular and systemic inflammation in melanoma patients and atherosclerotic *Ldlr*^-⁣/-^ mice. While short-term ICI therapy did not significantly alter arterial inflammation in melanoma patients, it enhanced plaque inflammation in mice, leading to more unstable lesions. Using hypercholesterolemic ApoE3*Leiden mice, Ewing *et al*. [173] proved that the co-stimulation of CD28-CD80/86 in T cells is crucial for the progression of accelerated AS following intervention and is modulated by CTLA-4-mediated co-inhibition. Inhibition of CD28-CD80/86 interactions by abatacept, an Ig fusion protein containing the extracellular domain of CTLA-4, markedly halted the progression of AS in hypercholesterolemic mice and reduced IFN-γ level, likely reflecting the decreased activation of CD4^+^ T cells. Furthermore, the application of CTLA-4 inhibitors significantly enlarged vascular lesions, confirming the regulatory role of CTLA-4 in AS progression [173].

In brief, the immune environment of atherosclerotic plaques is characterized by T cell and macrophage infiltration. Upregulation of PD-L1 on endothelial cells may represent a compensatory mechanism to limit inflammation. While a preclinical study has demonstrated ICIs to be pro-atherosclerotic [174], their precise impact on AS in cancer patients remains controversial. PD-1/PD-L1 or CTLA-4 inhibitors can enhance T cell response to APCs, leading to aggravated immune infiltration. Effector T cells can react to certain antigens in the plaques and exacerbate AS, while increased production of pro-inflammatory cytokines such as IFN-γ and TNF-β polarize macrophages toward the M1 phenotype, contributing to necrotic core formation and plaque instability.

### 3.7 ICI Therapy-Related Thromboembolism

Malignancies are associated with a higher risk of venous thromboembolism (VTE) and arterial thromboembolism (ATE). The application of chemotherapies and immune therapies can further increase the risk. In a cohort study by Kewan *et al*. [175], 10.5% of cancer patients treated with ICIs developed VTE. According to the information given by Roopkumar *et al*. [176], the incidence of VTE is 24% in patients on immunotherapy, and is related to a decreased overall survival. Therefore, the study on the correlation between ICIs and thrombotic events is non-negligible. Since ATE is closely related to AS, which we have elaborated on in previous paragraphs, we will focus on VTE in this unit.

Malignancies are a prothrombotic factor. The main initiator of coagulation, tissue factor (TF), is upregulated in tumor cells than normal adjacent cells, and high expression of TF is associated with poor differentiation and a higher risk of VTE [177]. Besides TF, cysteine protease, which can activate factor X and fibrinolysis proteins such as urokinase-type plasminogen activator A (u-PA), tissue-type plasminogen activator, and plasminogen activator inhibitors 1 and 2 (PAI-1 and PAI-2) can all be produced by tumor cells [178]. It’s also been reported that membrane-derived extracellular vesicles released by tumor cells, especially exosomes, microvesicles, and apoptotic vesicles, present procoagulant and immunogenic properties [179]. These vesicles can also be released by DCs and monocytes, which may distribute through the circulation system and cause distant thrombosis. Inflammatory cytokines like TNF-α and IL-1β secreted by tumor cells and the subsequent immune cell activation can both promote coagulation, and endothelial cells can be stimulated by cytokines to produce PAI-1, establishing a hypercoagulable environment [178, 180]. The adhesion of tumor cells to endothelial cells may also contribute to thrombus formation. It can be inferred that the combined effect of carcinoma and enhanced immune response in the presence of ICIs can further exacerbate the hypercoagulable status.

Sato *et al*. [181] validated the association between bleeding or clotting complications and anti-PD-1/PD-L1 therapies and pointed out that T cell activation can promote PD-L1^+^ CD14^+^ monocytes to express TF, triggering disorders in coagulation-fibrinolysis system. In conclusion, ICIs activate both T cells and APCs, inducing TF production and contributing to coagulation.

**Fig. 5.**
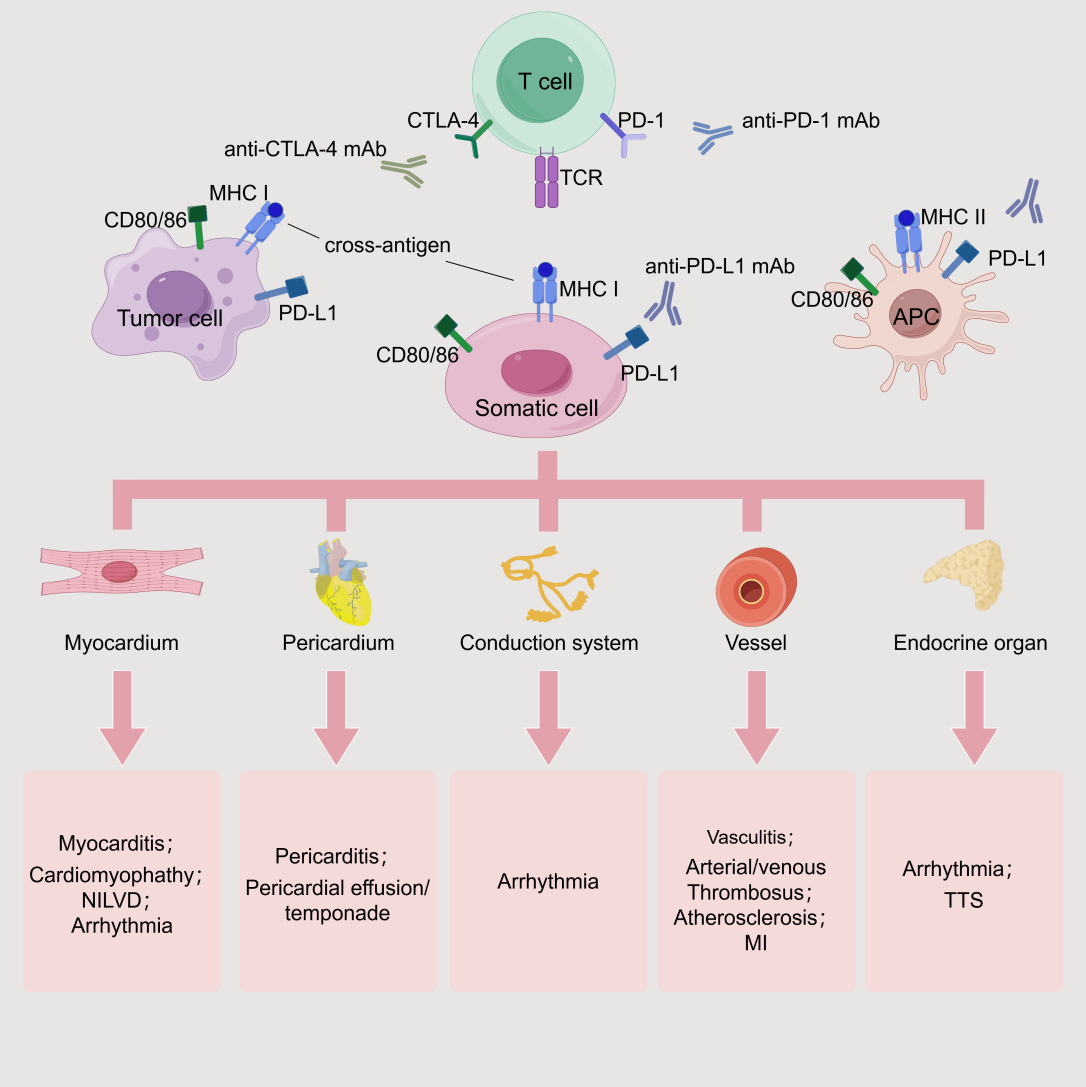
**ICI-related cardiovascular toxicities**. TCR can recognize the shared MHCs on tumor cells, somatic cells, and APCs. Depending on the targeted somatic cells and tissues, corresponding toxic reactions may occur. If affecting the myocardium, it may cause myocarditis, cardiomyopathy, arrhythmia, and NILVD. If the pericardium is involved, it may cause pericarditis and pericardial effusion. When the conduction system is affected, arrhythmia can be induced. If the vascular system is targeted, it can lead to vasculitis, thrombosis, AS, and even myocardial infarction. When the endocrine system is attacked, it may cause arrhythmia and TTS. mAb, monoclonal antibody; MI, myocardial infarction. This figure was created by figdraw (https://www.figdraw.com/#/).

In summary, ICI-induced cardiovascular toxicity represents a complex mechanism and process, typically involving the concerted action of multiple cell types and cytokines. Fig. 5 outlines the basic principles and types of toxicity associated with ICI-induced cardiovascular effects. Table 1 lists the key cells, cytokines, and primary mechanisms involved in various diseases.

**Table 1.** **Summary of ICI-induced cardiovascular toxicities: primary cellular drivers, responsible cytokines/mediators, and primary mechanism**.

ICI-associated cardiotoxicity	Primary cellular drivers	Cytokines/Mediators	Primary mechanisms
Myocarditis	CD8^+^ T cells, CD4^+^ T cells (Th1, Th17), B cells, neutrophils	IFN-γ, TNF-α, IL-17	- Autoimmune targeting of cardiac antigens leading to CD8^+^ T cell infiltration of the myocardium
			- IgG deposition around cardiomyocytes
			- Neutrophil infiltration of the myocardium
Pericardial diseases	CD4^+^ and CD8^+^ T cells, macrophages, B cells	DAMPs	- T cell-mediated pericardial inflammation
Arrhythmias	CD4^+^ T cells, CD8^+^ T cells, macrophages	IFN-γ, TNF, IL-2, IL-6, IL-10, IL-17	- T cell and macrophage infiltration of the cardiac conduction system
			- ICI-induced electrolyte imbalance
Heart failure	Macrophages	Catecholamines (in TTS)	- NILVD:
			■ IgG and C3 deposition around cardiomyocytes
			- TTS:
			■ Macrophage infiltration of the myocardium
			■ Catecholamine-mediated toxicity of the myocardium
Vasculitis	CD4^+^ T cells (Th1, Th17), macrophages, MGCs, α-SMC myofibroblasts, DCs	IL-6, IL-7, IL-9, IL-15, IL-17, IL-21, IFN-γ, IL-1β, TGF-β, MGF, TNF-α	- GCA: Formation of granulomatous infiltrates and damages the vascular wall
			- AAVs: Activated Th17 cells secrete IL-17 inducing neutrophil activation
			- LCV: deposition of circulating antigen-antibody complexes in blood vessels activates the complement system and subsequently recruits neutrophils
AS	CD8^+^ T cells, CD4^+^ T cells, macrophages, DCs	IL-18, IL-12, IFN-γ, TNF-α	- T cell and macrophage infiltration of atherosclerotic plaques leading to plaque destabilization and necrotic core expansion
Thromboembolism	Tumor cells, DCs, monocytes	TF, TNF-α, IL-1β	- Release of procoagulant microvesicles from tumor cells. DCs, and monocytes

DAMPs, damage-associated molecular patterns; LCV, leukocyte-crushing vasculitis; AAVs, anti-neutrophil cytoplasmic antibody (ANCA)-associated vasculitides; GCA, giant cell arteritis; TF, tissue factor.

## 4. Advances in Treatments for Cardiovascular irAEs

**Fig. 6.**
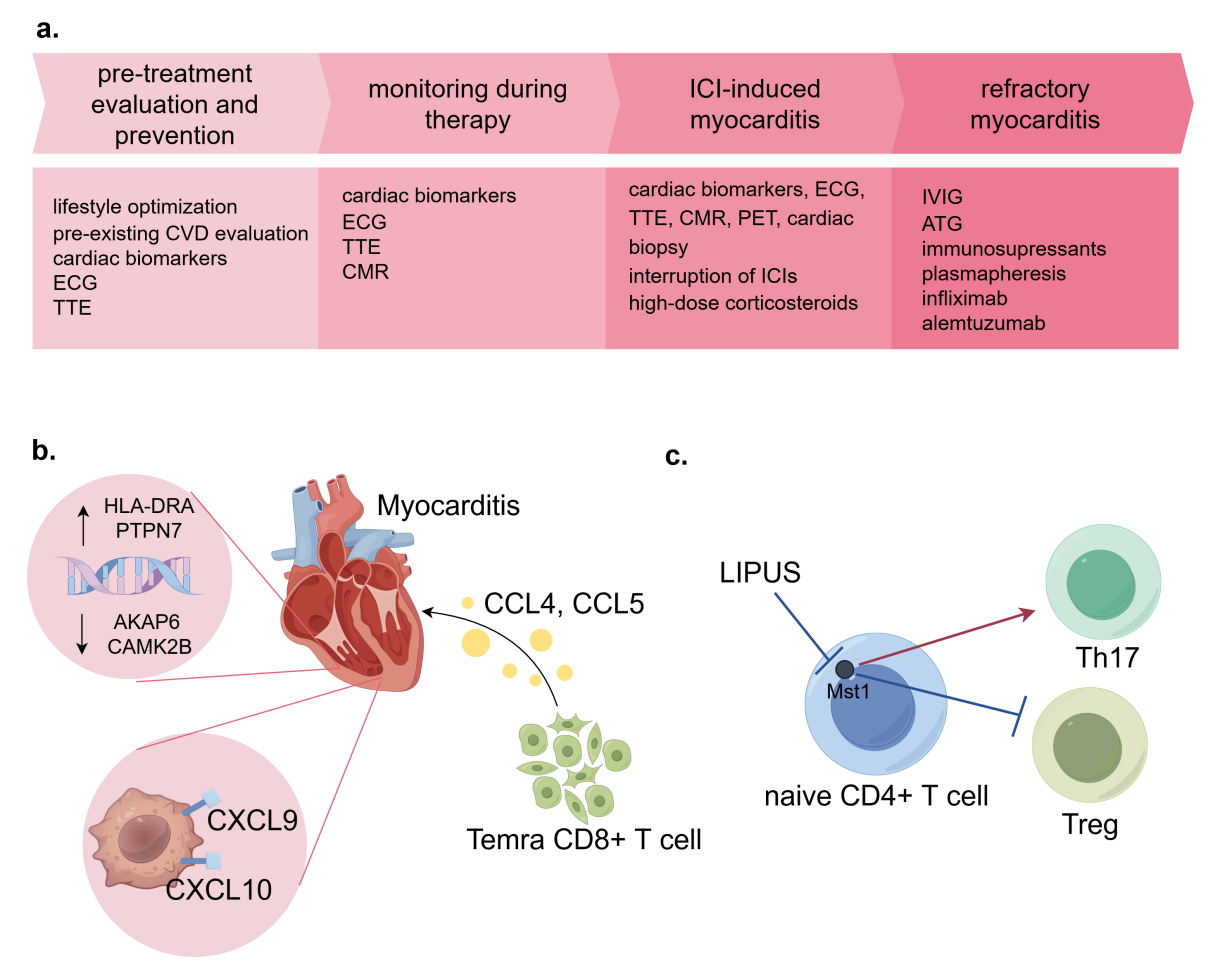
**Common strategies for ICI-induced cardiovascular toxicities and latest advances**. (a) Management of ICI-induced myocarditis. All patients should receive pre-treatment evaluation and take prevention methods before ICI therapy. Surveillance in on-going therapy is conducted via lab tests and imaging. Once myocarditis is suspected, a thorough evaluation is required including PET and cardiac biopsy. High-dose corticosteroids are recommended as first-line treatments. For refractory myocarditis, immunosuppressants and other treatments can be applied. (b) Advances in targets for ICI-induced myocarditis. Tissue in myocarditis presents higher HLA-DRA and PTPN7, and lower AKAP6 and CAMK2B expression. CXCL9^+^/CXCL10^+^ macrophages are abundant in myocarditis tissue. Temra T cells secret immune cytokines CCL4 and CCL5, which are potential therapeutic targets. (c) The potential of LIPUS in modulating autoimmune inflammation. LIPUS treatment can affect CD4^+^ T cell differentiation by inhibiting Mst1. CVD, cardiovascular disease; ECG, electrocardiogram; TTE, transthoracic echocardiography; CMR, cardiac magnetic resonance; PET, positron emission tomography; LIPUS, low-intensity pulsed ultrasound; IVIG, intravenous immunoglobulin; ATG, anti-thymocyte globulin; PCSK9 inhibitors, proprotein convertase subtilisin/Kexin type 9 inhibitors. This figure was created by figdraw (https://www.figdraw.com/#/).

At present, the diagnosis and treatment of cardiovascular irAEs are highly dependent on previous experience. According to the novel cardio-oncology guideline, early electrocardiogram (ECG), transthoracic echocardiogram (TTE), and cardiac biomarker monitoring are recommended for all patients planning to receive ICI therapy, as a primary prevention strategy [182] (see Fig. 6a). For patients who have already developed cardiovascular toxicities, whether or not to continue ICI therapy depends on the evaluated future benefits for patients. Treatments for the complications are highly similar to treatments of non-ICI-related cardiovascular toxicities, with the application of high-dose corticosteroids and immunosuppressants [183, 184]. Current approaches may be ineffective in some refractory cases, such as steroid-resistant myocarditis. Therefore, new advances in therapeutic targets for cardiovascular toxicities are urgently needed.

For patients who do not respond to glucocorticoids, alternative therapies may be considered [185], which include intravenous immunoglobulin [186], mycophenolate [187, 188, 189], anti-thymocyte globulin [190], plasmapheresis, infliximab, and alemtuzumab (an anti-CD52 monoclonal antibody). Despite the potential of these medications, the evidence supporting their effectiveness remains limited. Furthermore, abatacept has been shown to effectively mitigate myocarditis, supported by clinical data [189, 191, 192] and results from animal experiments [81, 85]. Additionally, abatacept has been reported to arrest the progression of accelerated AS in hypercholesterolemic mice, suggesting a broader therapeutic potential.

As previously mentioned, α-myosin, cardiac troponins, and some mitochondrial components are potential antigens for ICI-induced myocarditis, and hence, detecting the cross-antigens between cardiovascular and tumor tissues might be beneficial to reduce the risk of developing complications. A comparative study of various myocarditis samples and normal heart tissue revealed upregulated immune-associated gene expression of HLA-DRA, PTPN7, TNFRSF14, TNFAIP2, etc., in myocarditis cases. On the other hand, genes involved in heart motion and conductive systems, such as AKAP6 and CAMK2B, are substantially decreased, suggesting possible targets for myocarditis [193]. Zhu *et al*. [194] compared the transcriptomes of peripheral blood from ICI-induced myocarditis patients and other irAEs, and unveiled an expansion in cytotoxic terminally differentiated CD8^+^ T cells (Temra CD8^+^ T cells) and an elevation in the pro-inflammatory cytokines, CCL4 and CCL5. These studies suggest that targeting novel chemokines like CCL4 and CCL5 might be a new approach to treat ICI-induced myocarditis. Additionally, Lou and colleagues [195] reported that S100 family proteins are upregulated in ICI-induced myocarditis, which is also elevated in tumor tissue. Thus, S100 family proteins are potential biomarkers in the surveillance of the equilibrium between the progression of tumors and irAEs [195]. Furthermore, subsets of macrophages or monocytes expressing CXCL9 and CXCL10 were observed in both *Pdcd1^-⁣/-^ Ctla4^+⁣/-^* mice and patients with ICI-induced myocarditis. Hence, targeting CXCL9^+^/CXCL10^+^ macrophages may also be an approach to treat myocarditis [30] (see Fig. 6b).

A study on the HIPPO-pathway core components in myocarditis CD4^+^ T cells reported that autoimmune reaction might be attenuated by low-intensity pulsed ultrasound (LIPUS) [183]. This may be attributed to LIPUS’s ability to inhibit Mst1, a crucial enzyme in the HIPPO signaling, the inhibition of which reduces the generation of Th17 and facilitates the differentiation of T_regs_ (see Fig. 6c). This study provides new insights into the application of LIPUS in cardio-oncology.

The progression of AS is significantly accelerated during ICI therapy; however, there is currently no specific treatment strategy to address this complication [185, 196]. Even though corticosteroids tend to attenuate the progression of atherosclerotic plaque, the adverse effects of long-term use can not be overlooked [174]. Similar to general AS, stains and proprotein convertase subtilisin/kexin type 9 (PCSK9) inhibitors are also effective for ICI-related AS [174, 196, 197]. Besides, PCSK9 inhibitors potentiate tumor response to ICI-therapy by elevating MHC I expression on tumor cells, enhancing cytotoxic T-cell infiltration, without additional side effects [197].

## 5. Conclusions

ICIs have had a broader range of applications and achieved encouraging progress in cancer treatments over the past decade. However, irAEs are becoming matters of concern in the use of ICIs, especially rare but fatal cardiovascular toxicities increasingly threatening the lives of patients on ICI therapies. Hence, to improve ICI strategies and reduce the incidence and mortality of cardiovascular adverse events, abundant studies have been conducted to uncover the mechanisms behind the occurrence and development of ICI-induced cardiovascular toxicities. In this review, we collected novel findings on five main immune checkpoint pathways and integrated advances in the mechanisms of ICI-related myocarditis, cardiomyopathies, pericarditis, arrhythmias, heart failures, AS, vasculitis, and thromboembolism. Current studies generally support the notion that ICI-induced cardiovascular toxicities result from the ICIs triggering autoimmune attack on the cardiovascular system. However, the mechanisms underlying different manifestations of toxicity remain distinct, which we have thoroughly analyzed above. Additionally, we concluded the up-to-date progress made on the treatments for ICI-induced cardiovascular toxicities.

Nevertheless, questions remain to be answered in this field: (a) other unknown immune checkpoints involved in tumor progression; (b) the effect of the interactions of immune checkpoints on ICI therapies; (c) unknown antigens involved in ICI-related myocarditis and other inflammatory diseases; (d) mechanisms behind subtypes of ICI-related cardiovascular toxicities such as heart failures and arrhythmias; (e) interactions behind combined therapies of different ICIs, ICIs and radiotherapies, ICIs and chemotherapies, etc.; (f) potential biomarkers to monitor the occurrence and progression of ICI-related cardiovascular toxicities; (g) precise medical targets against ICI-induced cardiovascular toxicities.

It can be predicted that in the foreseeable future, the application of ICIs will continue to grow due to the relatively low incidence of severe adverse events. The establishment and development of cardio-oncology mark the importance scientists have attached to ICI-related cardiovascular toxicities. This multidisciplinary cooperation will bring better outcomes for ICI-treated patients.

## Abbreviations

AAV, anti-neutrophil cytoplasmic antibody-associated vasculitis; AF, atrial fibrillation; Akt, protein kinase B; AMA-M2, anti-mitochondrial antibody-M2; ANT, anti-adenine nucleotide translocator; AS, atherosclerosis; ATE, arterial thromboembolism; C3, complement 3; CEACAM-1, carcinoembryonic antigen-related cell adhesion molecule; CMR, cardiac magnetic resonance; CMTM6, CKLF-like Marvel transmembrane structural domain 6; CTLA-4, cytotoxic T lymphocyte-associated molecule-4; cTn, cardiac troponin; DAMP, damage-associated molecular patterns; DC, dendritic cell; DCM, dilated cardiomyopathy; ERK, extracellular regulated protein kinases; ERP, effective refractory period; GAL-3, galectin-3; GAL-9, galectin-9; GCA, giant cell arteritis; GPA, granulomatous polyangiitis arteriosa; HCM, human cardiac myosin; HF, heart failure; HMGB1, high mobility group box 1; ICI, immune checkpoint inhibitor; ICRS, individual case safety reports; IgV, immunoglobulin variable region; IgC, immunoglobulin constant region; irAEs, immune-related adverse events; ITSM, immunoreceptor tyrosine-based switch motif; LAG-3, lymphocyte activation gene-3 protein; Lck, lymphocyte-specific protein tyrosine kinase; LIPUS, low-intensity pulsed ultrasound; mAb, monoclonal antibody; mDC, myeloid dendritic cell; MDSC, myeloid-derived suppressor cell; MEK, mitogen-activated protein kinase/extracellular signal-regulated kinase (MAPK/ERK) kinase; MGC, multinucleated giant cell; MGF, macrophage growth factor; Mø, macrophage; Mt, mitochondria; mTOR, mammalian target of rapamycin; NET, neutrophilic extracellular traps; NILVD, non-inflammatory left ventricular dysfunction; PAI, plasminogen activator inhibitor; PCSK9, proprotein convertase subtilisin/kexin type 9; PD-1, programmed cell death receptor-1; PD-L1, programmed cell death ligand-1; PI3K, phosphatidylinositol-3-kinase; PLCγ, phospholipase Cγ; PNUTS, serine/threonine-protein phosphatase 1 regulatory subunit 10; PVR, poliovirus receptor; RAS, ras protein; ROS, reactive oxygen species; SHP-2, src homology region 2 domain-containing phosphatase-2; TDLNs, tumor-draining lymph nodes; TF, tissue factor; Th, helper T cell; TIGIT, T cell immunoglobulin and ITIM domain; TIL, tumor-infiltrating lymphocytes; TIM-3, T-cell immunoglobulin- and mucin-domain-containing molecule; TME, tumor microenvironment; T_reg_ cell, regulatory T cell; TTS, takotsubo syndrome; u-PA, urokinase-type plasminogen activator A; Vav1, vav guanine nucleotide exchange factor 1; VTE, venous thromboembolism; ZAP70, zeta chain-associated protein kinase 70kDa.
